# Trajectories of the relationships of physical activity with body composition changes in older men: the MrOS study

**DOI:** 10.1186/s12877-017-0506-4

**Published:** 2017-06-05

**Authors:** Deepika R. Laddu, Peggy M. Cawthon, Neeta Parimi, Andrew R. Hoffman, Eric Orwoll, Iva Miljkovic, Marcia L. Stefanick, for the Osteoporotic Fractures in Men Study Research Group

**Affiliations:** 1 Department of Physical Therapy, University of Illinois at Chicago, Chicago, Illinois 60612 USA; 2 California Pacific Medical Center Research Institute, San Francisco, California, USA; 3 Department of Epidemiology and Biostatics, University of California, San Francisco, CA USA; 4 Division of Endocrinology, Gerontology and Metabolism, Department of Medicine, Stanford University, Palo Alto, California, USA; 5 Department of Medicine, Oregon Health & Sciences University, Portland, OR USA; 6 Department of Epidemiology, University of Pittsburgh, Pittsburgh, PA USA; 7 Stanford Prevention Research Center, Department of Medicine, Stanford University, Stanford, California, USA

**Keywords:** Physical activity, Lean mass, Fat mass, Older men, Trajectories

## Abstract

**Background:**

Excess adiposity gains and significant lean mass loss may be risk factors for chronic disease in old age. Long-term patterns of change in physical activity (PA) and their influence on body composition decline during aging has not been characterized. We evaluated the interrelationships of PA and body composition at the outset and over longitudinal follow-up to changes in older men.

**Methods:**

Self-reported PA by the Physical Activity Scale for the Elderly (PASE), clinic body weight, and whole-body lean mass (LM) and fat mass, by dual-energy x-ray absorptiometry (DXA), were assessed in 5964 community-dwelling men aged ≥65 years at baseline (2000–2002) and at two subsequent clinic visits up until March 2009 (an average 4.6 and 6.9 years later). Group-based trajectory modeling (GBTM) identified patterns of change in PA and body composition variables. Relationships of PA and body composition changes were then assessed.

**Results:**

GBTM identified three discrete trajectory patterns, all with declining PA, associated primarily with initial PA levelshigh-activity (7.2% of men), moderate-activity (50.0%), and low-activity (42.8%). In separate models, GBTM identified eight discrete total weight change groups, five fat mass change groups, and six LM change groups. Joint trajectory modeling by PA and body composition group illustrated significant declines in total weight and LM, whereas fat mass levels were relatively unchanged among high-activity and low-activity-declining groups, and significantly increased in the moderate-activity-declining group.

**Conclusion:**

Although patterns of change in PA and body composition were identified, groups were primarily differentiated by initial PA or body composition rather than by distinct trajectories of change in these variables.

**Electronic supplementary material:**

The online version of this article (doi:10.1186/s12877-017-0506-4) contains supplementary material, which is available to authorized users.

## Background

Significant weight loss, particularly substantial lean mass loss [1], and fat mass gain have been identified as risk factors for chronic diseases in aging men [1–4]. Muscle mass has been reported to decline 1–3% per decade in men starting by age 30 and 2–3.3% per decade after age 60 [3, 5]. Progressive skeletal muscle loss accompanied by reduced muscle strength and declining functional performance [4, 6, 7], referred to as sarcopenia [6], leads to frailty and physical and mobility disabilities. Concomitant gains in fat mass also contribute to the reduced physical function and quality of life. [8].

Participation in regular physical activity (PA) has been shown to promote favorable changes in body composition, including prevention of lean mass loss and reduced fat mass gain [9–12]. Yet, PA generally decreases with age and only 37.8% of men aged 65–74 years report participating in the recommended minimum of 30 minutesmin of moderate-intensity physical activity on 5 or more days per week [13, 14]. The relationship of declining PA to lean mass loss or inability to retain lean mass may play an important role in the functional limitations that arise with aging.

Epidemiological evidence supports the hypothesis that higher regular PA and/or increasing PA levels attenuate total and fat weight gain through middle age [15, 16]. Substantial literature also suggests that age-related changes in body composition precede the gradual decline in PA as men age [1, 11, 17]. We are unaware of an assessment of both PA and body-composition changes or trajectories of these changes over an extended period in older men.

The long-term follow-up of the Osteoporotic Fractures in Men Study (MrOS) cohort provided the opportunity to apply innovative, methodological advances in the study of developmental trajectories [18, 19] to assess patterns of change in PA behavior during aging, taking into consideration baseline (routine) PA. Using a finite mixture modeling strategy to classify distinctive groups of older men based on similarities between their baseline and changes in PA, we estimated the proportion of men who demonstrated distinct patterns of PA change over an average 6.9 year follow-up. By relating the probability of group membership to individual’s PA, we can evaluate how PA changes may relate to changes in body composition (i.e., fat and lean masses) patterns as older men age. Finally, we applied joint (dual) trajectory models to analyze the correlation between the PA and body composition trajectories that were evolving contemporaneously as men aged, and to describe the development of the PA-body composition relationship over time. Additionally, multi-trajectory models, which were used to illustrate distinct patterns in the joint trajectories of PA and body composition, provides a compact approach for summarizing the within-individual correspondence of longitudinal data of PA and body composition measurements which other methods are unable to capture.

## Methods

### Participants

From March 2000 through April 2002, 5994 men, aged 65 years and older, enrolled in and completed baseline assessments in the multicenter MrOS study [20]. Recruitment was primarily through community-based mailing lists to six-geographic regions in the United States: Birmingham, AL; Minneapolis, MN; Palo Alto, CA; Pittsburgh, PA; Portland, OR; and San Diego, CA [20, 21]. Exclusions included bilateral hip replacement or inability to walk without the assistance of another person. A second and third clinic visit (V2 and V3) were conducted an average of 4.6 years and 6.9 years after baseline, respectively. Men who died or terminated were included in the trajectory building as long as they contributed at least one visit with PASE data before death or termination. For example, if they contributed PASE data at V1 and V2 and died after V 2, then their V1 and V2 values contributed to the trajectory model. Thus, this report of trajectory models was restricted to 5964 men who provided self-report data for physical activity (PA) by the Physical Activity Scale for the Elderly (PASE) and had a DXA measurement at one or both follow-up visits (Additional file 1: Figure S1). However, in models reporting absolute change in body composition trajectories relative to PA trajectories, men with loss to follow up due to deaths, termination or missing values were excluded from analyses. Thus, in absolute change models, only men who retuned for visit 3 and had non missing values for the body composition and PASE score were included change analyses**.** The total sample of men with visit 1 and visit 3 non missing measures of body weight: *n* = 3894, lean mass: *n* = 3641, and fat mass: *n* = 3641. The institutional review board at each center approved the study protocol and written informed consent was obtained from all participants.

### Physical activity measurements

PASE [22], developed specifically for community-dwelling older adults, was self-administered at baseline (V1), V2, and V3. PASE measures total, occupational, household, and leisure physical activities over the previous 7 days and asks about the intensity, frequency, and duration of a variety of activities, including walking; strenuous, moderate and light sports; muscle strength and endurance training; occupational activities, including standing or walking; lawn work and gardening; caring for another person; home repairs; and heavy and light housework. The frequency and duration of each activity is multiplied by an empirically derived item weight and summed to compute the total PASE score activity [22]. PASE scores, which have no units, provide a relative rather than absolute measure of PA levels. PASE has been previously validated with objective measures of physical activity [22–24], and has high test–retest reliability [22, 23].

### Assessment of weight, and body composition

Body weight was recorded with a balance beam scale at all but the Portland MrOS clinical site, which used a digital scale. Whole-body fat mass and lean mass (LM) were measured from whole-body scans using fan beam DXA scanners (QDR 4500 W, Hologic, Inc., MA), following a standardized procedures. All scan analyses were executed by certified DXA technicians. Reproducibility was ensured by use of a central quality control lab (San Francisco Coordinating Center). Quality assurance was carried out using a Hologic whole-body phantom, scanned repeatedly at each site to monitor reproducibility of measurements, and to calculate and apply appropriate correction factors to adjust for longitudinal drift in DXA measures.

### Questionnaire data

At baseline, participants reported their age, race/ethnicity, education, smoking status, alcohol use, and health status. Self-reported medical history data included history of physician diagnosis of diabetes mellitus, hypertension, coronary heart disease, congestive heart failure, chronic obstructive lung disease, and non-skin cancer.

### Statistical analysis

Group-based trajectory modeling (GBTM) was applied using TRAJ software in STATA (v. 9.2) which identifies clusters of individuals following similar progressions of a specified phenotype over some measure of time (e.g.; age, follow up time). [19] GBTM are descriptive analyses that identify latent groups of individuals based on patterns (i.e., decline, incline, cyclic), while simultaneously accounting for unobserved heterogeneity in the data [18]. Data analysis proceeded through three steps. The maximum number of groups (trajectories) for each phenotype was decided a priori. A quadratic shape of the pattern of change per trajectory was specified for all models, as recommended by Jones et al. 2001 [18, 19]. The number of trajectories were then fit sequentially until the most parsimonious model was determined by either the maximum number of groups decided by the Bayesian Information Criterion (BIC) or a priori. Briefly, the best fit model and number of trajectory groups were determined using the 2∆BIC > 10; such that subsequent complex models with an increased number of trajectories groups would not identify unique clusters of individuals. If the number of trajectories was greater than the maximum number of trajectories decided a priori then the models were fit with the number of trajectories decided prior to the analysis. After the final model was defined, mean posterior probabilities were calculated to ensure internal reliability of the model. In all models, an individual was assigned to a group based on his highest posterior group probability over the follow up. For generalizability, we only included specific groups comprised of at least 1% of the analytic cohort. The same model-fit criteria were repeated to determine trajectories of PA, total body weight and fat mass, and LM, over the maximum follow up, in separate models.

Joint trajectory modeling was employed to examine combinations of PA groups with each of the body composition variables (i.e., PA-weight, PA-fat mass, PA-LM) using similar model fit criteria. Baseline characteristics of participants were reported by PA trajectory groups as means, medians and proportions as appropriate, and differences between PA trajectory groups were tested using ANOVA and χ^2^ for continuous and categorical characteristics, respectively. Formal statistical tests, restricted to those who returned to visit 3 and had non missing measures at baseline and at visit 3, were performed to determine:

1. whether changes from baseline (V1) to visit 3 in body composition measures were statistically significant from zero within each trajectory, without consideration of the PA trajectories. This test will determine if GBTM correctly (and significantly) described the trajectory’s pattern (i.e., decline, incline, cyclic).
2. whether mean change in each body composition measure (computed [V3-V1] for each body composition trajectory) was significantly different with-in and across PA trajectories. Kruskal-Wallis *P*-values were reported due to slightly skewed body composition data. Tests of statistical significance were 2-tailed, with α = 0.05.

## Results

### Subject characteristics

PA trajectory patterns, all of *declining* PA, were identified by GBTM methods: high (baseline) activity, moderate activity, and low activity, representing 7.2%, 50.0%, and 42.8%, of the total analytic cohort, respectively. Descriptive statistics of the 5964 men in the cohort are presented in Table 1 by these PA (PASE score) trajectory groups. The average baseline age (SD) was 73.7 (5.9) years. The majority were Caucasian (89.4%). A greater proportion of men in the low-activity-declining group reported having hypertension, diabetes, CVD and CHD and past-year fall history compared to men in the moderate or high activity baseline groups (all *P* < .05).

**Table 1:** Baseline Characteristics by Physical Activity Groups in 5964 MrOS Men

	PA trajectories (PASE score)				
Characteristics	Low- activity-declining (*N* = 2555)	Moderate- activity- declining (*N* = 2977)	High- activity- declining (*N* = 432)	Whole cohort (*N* = 5964)	*P*
Change in PA^a^	−54.1 (45.7)	−58.5 (50.1)	−69.0 (56.9)	−57.6 (49.1)	<0.0001^b^
Mean age (y)	73.5 (5.8)	73.8 (5.9)	73.5 (6.2)	73.7 (5.9)	0.21
Age Category					0.37
≤ 73 yrs	1373 (53.7)	1544 (51.9)	238 (55.1)	3155 (52.9)	
> 73 yrs	1182 (46.3)	1433 (48.1)	194 (44.9)	2809 (47.1)	
Race/ethnicity (%)					<0.0001
White	2253 (88.2)	2699 (90.7)	382 (88.4)	5334 (89.4)	
African American	134 (5.2)	100 (3.4)	8 (1.9)	242 (4.1)	
Asian	96 (3.8)	83 (2.8)	12 (2.8)	191 (3.2)	
Hispanic	42 (1.6)	59 (2.0)	25 (5.8)	126 (2.1)	
Other	30 (1.2)	36 (1.2)	5 (1.2)	71 (1.2)	
College education or higher	1364 (53.4)	1579 (53.0)	232 (53.7)	3175 (53.2)	0.95
Alcohol intake (drinks/wk)					0.05
None	957 (37.5)	1006 (33.8)	142 (32.9)	2105 (35.3)	
Light	641 (25.1)	789 (26.5)	112 (25.9)	1542 (25.9)	
Moderate/high	952 (37.3)	1179 (39.6)	178 (41.2)	2309 (38.8)	
Smoking (*n*, %)					0.0004
None/past smoker	2441 (95.6)	2891 (97.1)	426 (98.6)	5758 (96.6)	
Current smoker	113 (4.4)	86 (2.9)	6 (1.4)	205 (3.4)	
Self-reported health (*n*, %)					<0.0001
Very poor/poor/fair	531 (20.8)	296 (9.9)	15 (3.5)	842 (14.1)	
Excellent/good	2022 (79.2)	2681 (90.1)	417 (96.5)	5120 (85.9)	
Height, mean, cm	174.4 (6.9)	174.0 (6.7)	173.6 (6.5)	174.1 (6.8)	0.03
Weight, mean, kg	84.5 (14.1)	82.2 (12.4)	80.8 (12.5)	83.1 (13.2)	<0.0001
BMI, mean kg/m^2^	27.8 (4.1)	27.1 (3.5)	26.8 (3.7)	27.4 (3.8)	<0.0001
BMI Categories (kg/m^2^)					<0.0001
< 25	640 (25.1)	861 (28.9)	138 (31.9)	1639 (27.5)	
25–30	1279 (50.1)	1540 (51.8)	231 (53.5)	3050 (51.2)	
≥ 30	636 (24.9)	574 (19.3)	63 (14.6)	1273 (21.4)	
History of CVD	799 (31.3)	747 (25.1)	77 (17.8)	1623 (27.2)	<0.0001
History of CHD	158 (6.2)	149 (5.0)	9 (2.1)	316 (5.3)	0.001
Self-reported cancer	749 (29.3)	871 (29.3)	115 (26.6)	1735 (29.1)	0.501
Self -reported Hypertension	1216 (47.6)	1214 (40.8)	133 (30.8)	2563 (43.0)	<0.0001
Self- reported Diabetes	362 (14.2)	268 (9.0)	18 (4.2)	648 (10.9)	<0.0001
History of Fracture after 50 yrs	585 (22.9)	655 (22.0)	110 (25.5)	1350 (22.7)	0.39
Fall in last year (*n*,%)	582 (22.8)	581 (19.5)	94 (21.8)	1257 (21.1)	0.01

Mean (SD) or proportion as shown

*BMI* body mass index, *CHD* coronary heart disease, *CVD* cardiovascular disease, *PA* physical activity

*P*-values indicates the overall difference among PA trajectories

^a^Change measured as change in PASE score from visit 1 to visit 3.

^b^Significance observed in PA change between high activity declining compared to moderate activity declining (*P* < .001) or compared to low activity-declining groups (*P* < 0.001); ANOVA

### Patterns of change in PA

**Fig. 1:**
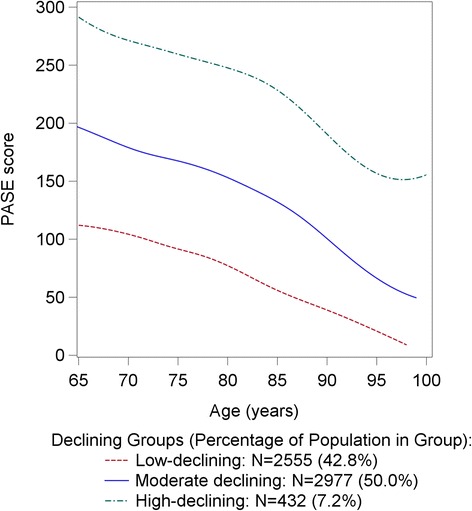
PA trajectories by age; total *N* = 5964; mean 6.9 year follow up. PA groups based on self-reported PA via PASE scores

As noted above, the final models showed a *decline* in PA for all three trajectory groups (*P* for change not zero <0.05); however, the decline in activity differed significantly among the three PA groups (*P* < .0001) over the 6.9 year follow-up (Table 1, Fig. 1), with the greatest declines observed in men in the high-activity-declining group (*P* for pairwise difference between high-activity-declining and low-activity-declining <.001; *P* for pairwise difference between high-activity-declining and moderate-activity-declining <.001).

### Individual patterns of change in body weight, LM, and fat mass

**Fig. 2:**
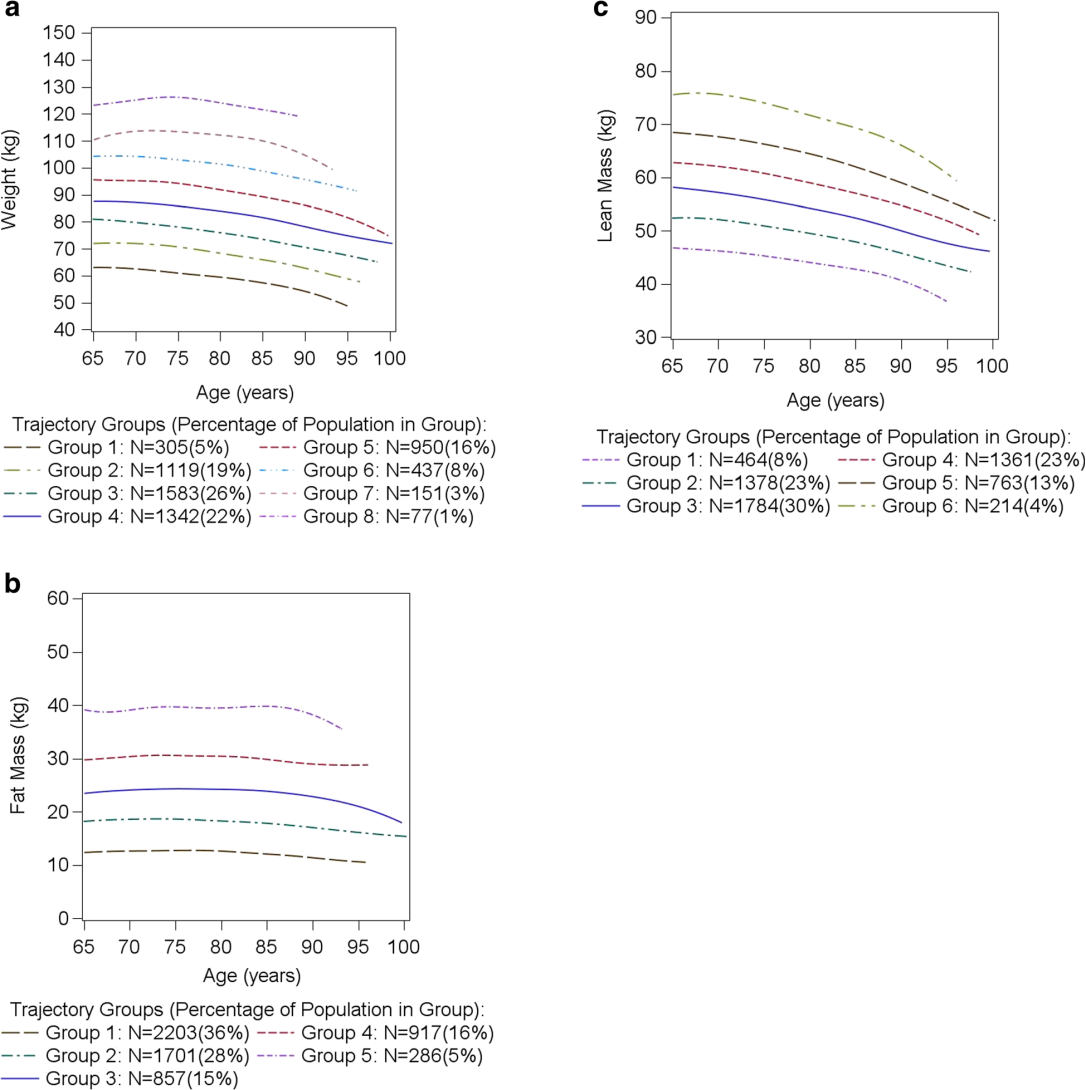
**a**–**c** Trajectories of body weight (**a**), Total body fat mass (**b**), and Lean mass (**c**). Number of groups reflect most parsimonious model

When changes of body weight, total body fat mass and LM were analyzed individually, there were eight body weight change groups, five fat mass change groups and six LM change groups in the most parsimonious models. Changes from baseline to visit 3 were shown to be declines in almost all eight weight groups and all six LM groups, with greater declines in body weight and LM among men with lower (trajectory 1) versus higher baseline weight or LM, respectively. Increases in fat mass were observed in two fat mass groups, and small, statistically significant declines were observed in one fat mass group from visit 1 to visit 3 (Additional file 1: Table S1, Fig. 2).

### Joint trajectory patterns of physical activity and body composition

**Fig. 3:**
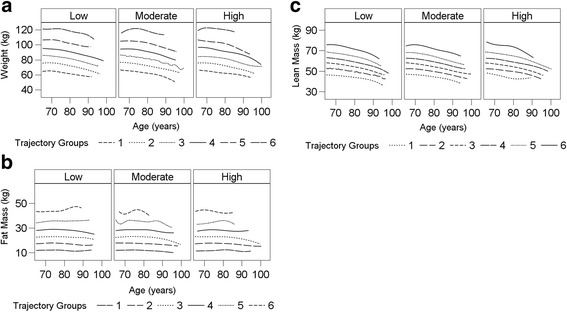
Joint Trajectory modeling of PA trajectories across body weight (**a**), Total body fat mass (**b**), and, Lean mass (**c**) using BIC criteria >410 with *p* < .05

Figure 3a–c graphically illustrates the joint trajectory models for changes in patterns of each body composition measure conditional on PA. For each joint trajectory model the most parsimonious models identified six trajectories for the body composition measure and three PA trajectories.

To determine whether body composition patterns differed significantly by PA trajectory, we computed the mean change (V3-V1) in the six groups of body weight, fat mass, LM groups, respectively, relative to the three declining PA trajectories (Table 2). We addressed whether: 1) changes in body composition measures from baseline to visit 3 were statistically significant from zero within each trajectory; 2) changes in body composition measures differed significantly within each PA trajectory; and, 3) changes in body composition measures from baseline to visit 3 were globally different across all three PA trajectories. Overall, body weight declined in most bodyweight/PA trajectory groups (*P*_V3-V1_ < .05 for most). Moreover, changes in body weight differed among the eighteen combinations of PA and body weight trajectories (overall ANOVA *P* < .0001). Within the low-activity-declining and moderate-activity-declining groups, mean body weight changes by the six body weight trajectories were significantly different from one another (*P* for ANOVA within activity declining groups <.0001). Conversely, there were no statistically significant differences in body weight change within the body weight trajectories for the high-activity-declining group (ANOVA within high activity group, *P* = .24).

**Table 2:** Mean Body Composition Changes from Baseline to Visit 3 by PA Trajectory in Older Men^a,b^

		Change in body weight by activity and weight pattern									
	Traj.	Low-activity-declining			Moderate-activity-declining				High-activity-declining		*P*_overall_^d^
		*N*	Mean (SD)	*P*_(ΔV3-V1)_	*N*	Mean (SD)	*P*_(ΔV3-V1)_	*N*	Mean (SD)	*P*_(ΔV3-V1)_	
Body weight Traj	1	207	−2.61 ± 4.76	<.0001	306	−2.30 ± 4.20	<.0001	55	−3.12 ± 4.84	0.0002	<.0001
	2	552	−2.38 ± 4.49	<.0001	982	−2.17 ± 6.15	<.0001	70	−1.23 ± 5.25	.09	
	3	613	−2.47 ± 5.83	<.0001	962	−2.32 ± 4.79	<.0001	138	−2.67 ± 4.57	<.0001	
	4	678	−2.80 ± 4.64	<.0001	593	−2.43 ± 4.35	<.0001	167	−2.05 ± 4.07	<.0001	
	5	275	−0.65 ± 6.76	.21	201	−2.00 ± 8.42	005	16	−2.44 ± 6.46	.24	
	6	111	1.00 ± 9.11	.37	23	33.35 ± 9.00	.19	15	−0.04 ± 5.50	.98	
*P*_within_ body weight-PA traj group^c^			<.0001			<.0001			.24		
		Change in fat mass by activity and fat mass pattern									
	Traj.	Low-activity-declining			Moderate-activity-declining			High-activity-declining			*P*_overall_^d^
		*N*	Mean (SD)	*P*_(ΔV3-V1)_	*N*	Mean (SD)	*P*_(ΔV3-V1)_	*N*	Mean (SD)	*P*_(ΔV3-V1)_	
Fat mass Traj.	1	1181	−0.19 ± 2.93	.07	619	−0.10 ± 3.23	.56	178	−0.01 ± 3.14	.96	<.0001
	2	900	0.05 ± 3.47	.72	757	0.33 ± 3.36	.05	127	0.08 ± 3.36	.83	
	3	394	−0.19 ± 2.64	.26	205	−0.38 ± 3.18	.20	100	−0.34 ± 2.36	.21	
	4	509	0.36 ± 4.33	.13	490	0.45 ± 4.13	.08	33	0.84 ± 2.98	.18	
	5	109	1.07 ± 5.22	.07	237	1.54 ± 4.72	.0004	16	1.66 ± 2.62	.08	
	6	8	−1.92 ± 8.30	.67	93	2.32 ± 4.98	.004	8	−0.35 ± 3.65	.84	
*P*_within_ fat mass-PA traj group^c^			<.0001			<.0001			.51		
		Change in LM by activity and LM pattern									
	Traj.	Low-activity-declining			Moderate-activity-declining			High-activity-declining			*P*_overall_^d^
		*N*	Mean (SD)	*P*_(ΔV3-V1)_	*N*	Mean (SD)	*P*_(ΔV3-V1)_	*N*	Mean (SD)	*P*_(ΔV3-V1)_	
LM Traj.	1	220	−2.14 ± 1.91	<.0001	226	−1.70 ± 2.31	<.0001	17	−2.29 ± 2.72	.014	<.0001
	2	581	−2.13 ± 2.61	<.0001	687	−1.66 ± 2.25	<.0001	114	−1.91 ± 2.27	<.0001	
	3	644	−2.17 ± 2.75	<.0001	972	−1.77 ± 2.23	<.0001	159	−2.14 ± 1.94	<.0001	
	4	596	−2.03 ± 3.14	<.0001	694	−1.79 ± 2.62	<.0001	76	−1.60 ± 2.31	<.0001	
	5	342	−1.74 ± 3.09	<.0001	360	−2.21 ± 3.20	<.0001	63	−0.93 ± 3.01	.04	
	6	128	−1.60 ± 4.36	.002	69	−1.98 ± 3.93	.002	16	−1.91 ± 3.77	.14	
*P*_within_ LM-PAtraj group^c^			.04			<.001			.81		

*∆* change, *LM* lean mass, *PA* physical activity, *SD* standard deviation, *Traj* trajectory, *V* visit

^a^Mean change (SD); P-val (ΔV3-V1), *P*-values for change in measure from baseline to visit 3 testing if change is significantly different than 0 within each joint-trajectory.

^b^Means reflect men who had non missing values at both, baseline and visit 3 for each measure.

^c^Significance observed in respective body composition trajectories *within* PASE trajectory; ANOVA

^d^Significance observed in respective body composition trajectories *across* PASE trajectory; ANOVA

Tests of statistical significance, *P* = 0.05

Total Men with visit 1 and visit 3 non missing measures: Body weight = 3894, Lean Mass = 3641, Fat Mass = 3641

Fat mass changes remained relatively unchanged (stable) across most fat mass/PA groups over time (*P*_V3-V1_ > .05 for most); though significant increases in fat mass were observed in three fat mass trajectories in the moderate-activity-declining group (*P*_V3-V1_ < .05). Changes in fat mass differed by the eighteen combinations of fat mass and PA patterns (overall ANOVA *P* < .0001). Within the low-activity-declining and moderate-activity-declining groups, significant differences in mean fat mass changes by the six fat mass trajectories were observed (*P* for ANOVA within activity-declining groups <.0001). However, the overall differences in fat mass change within the fat mass trajectories for the high-activity-declining group were not significant (ANOVA within high activity group, *P* = .51).

LM declined in most of the LM/activity pattern groups (*P*_V3-V1_ < .05 for most). Changes in LM differed by the eighteen combinations of LM and activity patterns (overall ANOVA *P* < .0001). Similar to the other body composition measures, there were differences in the mean changes in LM by LM trajectories within the low-activity-declining and moderate-activity-declining groups, (*P* for ANOVA within activity declining groups <.05). Additionally, there were no significant differences in mean LM changes within the LM trajectories for the high-activity-declining group (ANOVA within high activity group, *P* = .81).

## Discussion

In this descriptive study, we modeled trajectories of changes in PA and body composition over a nearly 7-year follow-up period with group based trajectory methods (GBTM). Three key findings emerged from these analyses. First, three discrete trajectories of PA change were identified among these older men, based primarily on baseline (regular) self-reported PA. All declined over the 7 year follow-up, however, the greatest declines were observed among men in the high-activity-declining group, compared to the moderate-activity- and low-activity-declining trajectories (both *P* < .001). Although the magnitude of the difference in change was modest it is important to recognize that men who report the highest PA levels at the outset of a longitudinal study may decrease PA much more than less active men over the course of the study. Nonetheless, these men still reported higher activity levels than the other two groups at subsequent visits. Second, greater declines in body weight and LM were observed among men with lower versus higher baseline body weight and LM, respectively. Joint-trajectory modeling showed that total body weight and LM declined, whereas fat mass levels were relatively unchanged among high activity- and low-activity-declining groups. In contrast, fat mass was significantly increased in three of the six moderate-activity-declining trajectories. Third, with-in group changes in body weight, fat mass and LM were associated with patterns of PA among low activity, and moderate activity declining men, but were not statistically different among high-activity-declining men.

Data on long-term PA changes in older men are scarce [11, 25, 26]. Previous studies of PA behavior in older adults have generally been cross-sectional [14, 27, 28] or based on self-reported baseline PA levels only and have not captured an individual’s change in activity over time. In the MrOS cohort, all trajectory modeling groups showed declines in PA levels, consistent with a previous MrOS report of decreased PA levels over a 5-year follow up, with the greatest PA declines attributed primarily to decreases in occupational, leisure, and household PA and poor health [24]. In contrast, de Souto et al. (2014) reported that total volume and duration of PA remained relatively stable in another cohort of older men; however, this was based on only 12- and 38 month follow ups [29].

To our knowledge, this is the first study to use latent class growth modeling analysis to describe, simultaneously, the relationship of changes in PA and changes in body-composition in older men. Examining the dynamic changes in PA as it relates to changes in body composition is particularly important in older adults, for whom the relation between PA and body composition may be the consequence of underlying poor health or chronic disease. Notably, a greater proportion of men in the low-activity-declining reported having debilitating health issues versus those in the moderate- and high-activity-declining groups (Table 1). Previous prospective studies showed a tendency toward lean mass loss and fat mass gain over time in healthy, weight-stable persons [26, 30]. In the Women’s Health Initiative (WHI) cohort of women aged 50–79, lean mass decreased across all age (decade) groups and all PA levels over about 6 years, whereas, fat mass change depended on both age and PA level, with higher PA levels attenuating fat gains in younger women (age 50–59), while fat loss was observed at older ages, regardless of PA level [9]. In contrast, in the MrOS cohort of men aged 65 and older, significant body weight and lean mass loss was observed in nearly all body weight and LM groups and across all PA change trajectories, whereas fat mass levels increased significantly among moderate-activity-declining groups, but remained relatively stable in high-activity- and low-activity-declining trajectory groups (*P* > .05) over nearly 7 years. Significant differences in the mean change of each body composition measure were observed among men with in low-activity-and moderate-activity-declining PA groups, only (Table 2; Fig. 3), suggesting that changes in body weight, fat mass and LM were associated with patterns of PA among low-activity-, and moderate-activity declining men, but not among high-activity-declining men. Nonetheless, our findings of symmetry in the declining patterns of weight loss and LM further support the hypothesis that there is a failure to conserve lean mass with weight loss in old age [4, 11, 25], whereas maintaining higher PA levels earlier in life and during aging may have a modest role in attenuating weight and lean mass declines in older men (Table 2, ANOVA within high activity group, both *P* > 0.05), consistent with the results reported by previous investigators [11, 25].

### Strengths & limitations

These analyses included repeated PA measures in a large cohort of elderly men, allowing the capture of distinct, individual changes in PA engagement in older adults, and employed DXA to assess longitudinal body composition changes, using validated methods that correlate highly with other criterion methods [26]. As MrOS participants were predominantly healthy Caucasians, further investigation of patterns in PA-body composition changes in racial and ethnic minorities and men with specific health conditions, is warranted. Also, trajectory modeling is inherently limited in capturing individual variability and may lead to over-grouping with significant variation within PA trajectories. Additionally, PASE scores do not measure actual activity levels or the metabolic equivalent of a task or minutes of exercise; therefore, these results cannot be readily interpreted in relationship to national recommendations for PA in older adults. Corroborating the reported declines in PA with objective measures, to further understand PA in the context of dose (intensity, frequency and duration), may be warranted. Adjusting for intensity and mode of exercise to demarcate resistance training was not feasible in this study. Lastly, given the observational nature of the study, we cannot infer causality as to whether declines in loss of weight and lean mass were the result of declines in PA or confounded by other demographic, lifestyle or genetic factors affecting PA and body composition changes. Some of the groups identified, especially in the joint PA/body composition models, are very small and there is limited statistical power to draw conclusions about such groups.

## Conclusion

The analyses from this longitudinal study of older men suggest that initial PA and body composition may differentiate PA and body composition changes over 6.9 years of follow-up.

## Additional file

Additional file 1: Figure S1. Flow Chart describing MrOS sample size for Trajectory Analysis. This figure describes, total sample size of men included from each study visit, and specifically, the sample of men with non missing values for the body composition and PASE score. A description of how the Proc Traj methods accommodates missing data into the trajectory building is also provided. **Table S1.** Declining Patterns of Body Composition Changes From Visit 1 to Visit 3 (2000 to 2009). This table reports the individual patterns of change in body weight, lean mass, and fat mass, according to the most parsimonious model of eight body weight trajectories, five fat mass trajectories groups and six lean mass trajectories, respectively. (DOCX 30 kb)

## Abbreviations

BIC: Bayesian Information Criterion
DXA: Dual energy x-ray absorptiometry
GBTM: Group-based trajectory modeling
LM: Lean mass
MrOS: Osteoporotic Fractures in Men Study
PA: Physical activity
PASE: Physical Activity Scale for the Elderly
SD: Standard deviation
V: Visit
