# Supplementary material for: Trajectories of the relationships of physical activity with body composition changes in older men: the MrOS study
Source: BMC Geriatr. 2017 Jun 5;17:119. doi: 10.1186/s12877-017-0506-4 (PMC5460414; doi:10.1186/s12877-017-0506-4)
Supplement: Additional file 1: Figure S1. — Flow Chart describing MrOS sample size for Trajectory Analysis. This figure describes, total sample size of men included from each study visit, and specifically, the sample of men with non missing values for the body composition and PASE score. A description of how the Proc Traj methods accommodates missing data into the trajectory building is also provided. Table S1. Declining Patterns of Body Composition Changes From Visit 1 to Visit 3 (2000 to 2009). This table reports the individual patterns of change in body weight, lean mass, and fat mass, according to the most parsimonious model of eight body weight trajectories, five fat mass trajectories groups and six lean mass trajectories, respectively. (DOCX 30 kb) [file 12877_2017_506_MOESM1_ESM.docx]

**Additional file 1**

Figure S1. Flow Chart describing MrOS sample size for Trajectory Analysis

109 Refused

568 Died (7 with missing visit 1 DXA measures)

88 Terminated (2 with missing visit 1 DXA measures)

Baseline Visit

N=5994 Men

Visit 2

4530 Clinic Visit

699 SAQ only

Men

1 missing status

85 Refused

447 Died

59 Terminated

Visit 3

3778 Clinic visit

118 Home only

741 SAQ only

Proc Traj accommodates data missing completely at random at different time points. All analyses included men with at least one measurement of PA (PASE score), and DXA body composition measures at baseline, V2 or V3 examinations. Men with loss to follow up due to deaths, termination or missing values were included in the trajectory building as long as they contributed at least one visit with PASE data before death or termination.

At visit 3, specifically men who had a clinic visit (which included body composition measurements) and were SAQ only, also had a PASE score assessed. Additionally, among the 118 men who had a home visit, only one man had non missing values for body composition measures. Overall, 30 participants were excluded because they had missing DXA at one or more visits; the reason for missingness was due to the following reasons: seven died before V2, two terminated participation in MrOS before V2, and three died before V3. Of the remaining, seven men were SAQ at V3, 2 were SAQ and a home visit at V3 and nine had missing body composition measures at all visits.

Notably, men with loss to follow up due to deaths, termination or missing values were not included in analyses examining absolute change in body composition trajectories relative to PA trajectories displayed in Table 2. Thus, only men who retuned for visit 3 and had non missing values for the body composition and PASE score were included change analyses. The total sample of men with visit 1 and visit 3 non missing measures: Body weight =3894, Lean mass=3641, Fat mass=3641.

Table S1. Declining Patterns of Body Composition Changes From Visit 1 to Visit 3 (2000 to 2009).^a,b^

|  | Body composition Measures mean change (SD) | | | | | | | | | | | | |
| --- | --- | --- | --- | --- | --- | --- | --- | --- | --- | --- | --- | --- | --- |
| Trajectories^c^ | Weight change (kg) | *P* | | Fat Mass  change (kg) | | | *P* | LM change (kg) | | *P* | | | |
| 1 | .30 (9.02) | .73 | .73 (.20) | | | .0002 | | -1.93 (2.26) | | | | <.0001 | |
| 2 | -2.58 (4.54) | <.0001 | -.25 (2.81) | | | .032 | | 1.95 (2.39) | | | | <.0001 | |
| 3 | -2.54 (4.48) | <.0001 | -.08 (3.06) | | | .33 | | -1.84 (2.37) | | | | <.0001 | |
| 4 | -2.81 (4.38) | <.0001 | .18 (3.56) | | | .01 | | -1.85 (2.81) | | | | <.0001 | |
| 5 | 1.50 (8.11) | .21 | 1.26 (5.19) | | | .002 | | -1.91 (3.15) | | | | <.0001 | |
| 6 | -1.77 (6.33) | <.0001 |  | |  | | | -1.77 (4.13) | | | | <.0001 | |
| 7 | -2.06 (5.42) | <.0001 |  | |  | | | |  | | | |  |
| 8 | -1.43 (7.19) | .0008 |  | |  | | | |  | |  | | |

LM, lean mass; SD, standard deviation

^a^ Mean change in body composition calculated as the difference from visit 1 to visit 3

^b^ *P* -values for change in measure from baseline to visit 3 significantly different than 0; *P* <.05

^c^ Trajectories represent the most parsimonious model for each body composition measure.
